# Nontargeted metabolomics-based multiple machine learning modeling boosts early accurate detection for citrus Huanglongbing

**DOI:** 10.1093/hr/uhac145

**Published:** 2022-06-27

**Authors:** Zhixin Wang, Yue Niu, Tripti Vashisth, Jingwen Li, Robert Madden, Taylor Shea Livingston, Yu Wang

**Affiliations:** Citrus Research & Education Center, Institute of Food and Agricultural Sciences, University of Florida, Lake Alfred, Florida 33850-2299, U.S.A; Department of Mathematics, University of Arizona, Tucson, Arizona 85721-0089, U.S.A; Citrus Research & Education Center, Institute of Food and Agricultural Sciences, University of Florida, Lake Alfred, Florida 33850-2299, U.S.A; Citrus Research & Education Center, Institute of Food and Agricultural Sciences, University of Florida, Lake Alfred, Florida 33850-2299, U.S.A; Citrus Research & Education Center, Institute of Food and Agricultural Sciences, University of Florida, Lake Alfred, Florida 33850-2299, U.S.A; Citrus Research & Education Center, Institute of Food and Agricultural Sciences, University of Florida, Lake Alfred, Florida 33850-2299, U.S.A; Citrus Research & Education Center, Institute of Food and Agricultural Sciences, University of Florida, Lake Alfred, Florida 33850-2299, U.S.A

## Abstract

Early accurate detection of crop disease is extremely important for timely disease management. Huanglongbing (HLB), one of the most destructive citrus diseases, has brought about severe economic losses for the global citrus industry. The direct strategies for HLB identification, such as quantitative real-time polymerase chain reaction (qPCR) and chemical staining, are robust for the symptomatic plants but powerless for the asymptomatic ones at the early stage of affection. Thus, it is very necessary to develop a practical method used for the early detection of HLB. In this study, a novel method combining ultra-high performance liquid chromatography/mass spectrometry (UHPLC/MS)-based nontargeted metabolomics and machine learning (ML) was developed for conducting the early detection of HLB for the first time. Six ML algorithms were selected to build the classifiers. Regularized logistic regression (LR-L2) and gradient-boosted decision tree (GBDT) outperformed with the highest average accuracy of 95.83% to not only classify healthy and infected plants but identify significant features. The proposed method proved to be practical for early detection of HLB, which tackled the shortcomings of low sensitivity in the conventional methods and avoid the problems such as lighting condition interference in spectrum/image recognition-based ML methods. Additionally, the discovered biomarkers were verified by the metabolic pathway analysis and content change analysis, which was remarkably consistent with the previous reports.

## Introduction

Huanglongbing (HLB), i.e. citrus greening, is one of the most destructive citrus diseases [1]. It is presumably brought about by three species of phloem-limited bacteria *Candidatus* Liberibacter, i.e. *Ca. L. americanus* (*Ca*Lam), *Ca. L. asiaticus* (*Ca*Las) and *Ca. L. africanus* (*Ca*Laf) [2]. The pathogens are vectored by psyllids or infected plant buds used in grafting. The vector psyllid *Diaphorina citri* carries *Ca*Lam and *Ca*Las, and *Trioza erytreae* transmits *Ca*Laf [2]. The discovery of *Ca*Lam and *Ca*Las in Brazil in 2004, and the establishment of *Ca*Las infection symptoms and confirmation of HLB in Florida state of the USA in 2005 are of great concern to the citrus industry [3, 4]. Continuing to spread worldwide, HLB destroyed thousands of acres of citrus trees, resulting in a decline in quality as well as the harvest of the fruits, with an immense loss of global citrus production. In Florida, the majority of citrus orchards (>95%) were negatively impacted by *Ca*Las [4]. Citrus production has declined more than 60% compared to 20 years ago, causing serious economic losses [5].

Citrus trees affected with HLB usually take 6 to 12 months to show noticeable symptoms. Typical symptoms include yellowing of leaves and shoots with blotchy mottles, bearing small, malformed and asymmetric fruits [6]. Symptoms spread throughout the tree through infected branches. For tree disease detection, the asymptomatic period is critical. However, it is challenging to distinguish the healthy trees from the infected but asymptomatic ones because there are not any noticeable differences in appearance. HLB research is hindered by the unculturability of pathogenic bacteria in artificial media [7]. Detection and identification of pathogens are also difficult, likely due to low concentrations as well as an uneven pattern found in host and carrier psyllids [8]. In recent years, the classical quantitative real-time polymerase chain reaction (qPCR) has been used as a common technique for the detection of HLB, but it is time- and labor-consuming and costly because of the need for specialized equipment and professionals. Moreover, it behaves powerlessly for the diagnosis of HLB within the asymptomatic period [9]. Pandey et al. [10] proposed a method of Coomassie Brilliant Blue Staining - Microscopy using psyllids feeding on the salivary sheaths present under the blue dots of leaves for early detection of *Ca*Las. Though simple, this method is low in sensitivity and requires specialized sectioning processing for samples. Multi-spectral or hyperspectral imaging technologies based on the unmanned aerial vehicle (UAV) system have been applied to detect HLB in the field, which can acquire relatively high accuracy of prediction, but they often involve multi-scale and high-dimensional spatial information, making it difficult to conduct effective application [11]. Diverse spectral methods, including mid-infrared spectroscopy [12], visible–near infrared spectroscopy [13], fluorescence imaging spectroscopy [14], Raman spectroscopy [15] and laser-induced breakdown spectroscopy [16] have also been applied for the detection of HLB combined with different pattern recognition algorithms. Some superficially acceptable results have been achieved, yet these methods usually use a single imaging sensor, and different physiological responses of trees cannot be detected, which are very important to determine the disease status of trees and to enhance detection ability [17]. In this case, it is of great necessity, though challenging, to develop a highly reactive and practical method used for the early detection of HLB.

The plant metabolome is the repertoire of small molecules that plants accumulate at specific time points. As the end products of cellular processes and genetic or environmental information processing, metabolites are directly linked to the phenotypes of plants, and their levels can be regarded as the ultimate response of biological systems to genetic or environmental changes [18]. In the presence of disease, the abundances of some metabolites in plants are significantly altered compared to the healthy states [19]. However, metabolites own a wide range of diverse chemical structures and are also highly dynamic in time and space, raising the challenges for analytical techniques in measuring them as a whole [20]. Currently, the most widely used technique in plant metabolomics is ultra-high performance liquid chromatography/mass spectrometry (UHPLC/MS). Compared to other alternatives, UHPLC/MS provides higher versatility in the preparation of samples and chromatographic separations [21]. It can accommodate almost all classes of compounds, while maintaining high speed and high sensitivity, in addition to providing accurate structural information. These advantages enable UHPLC/MS an exceptional functional choice dealing with nontargeted metabolomics, i.e. unbiased measurements of metabolic profiles involving identifying hundreds to thousands of metabolites and exposing their level changes [22]. Machine learning (ML) is an automated modeling approach to data analysis. This idea that systems are capable of acquiring knowledge and recognizing patterns, even making choices, founded the theory of artificial intelligence. Numerous ML algorithms are known to the public, but it is only until recent times that scientist find the ability to automatically apply complicated mathematical calculations to big data in science researches [23]. In the agricultural area, the development of supervised ML algorithms, a set of analytical methods that train and validate models, optimize parameters on labeled datasets, and iteratively learn from data to gain insights provides a more powerful and efficient idea by which not only healthy and infected plants can be classified, but molecular signatures like important metabolites involved in plant-pathogen interactions can also be identified. Sankaran et al. [13] developed an HLB detection system using visible-NIR spectroscopy and compared quadratic discriminant analysis (QDA), k-nearest neighbor, linear discriminant analysis (LDA), and software-independent classification analogy modeling (SIMCA). Algorithms based on SIMCA and QDA had the highest accuracy among all methods. Wetterich et al [14] used two ML algorithms, the support vector machine (SVM) and the artificial neural network (ANN), to differentiate HLB and zinc deficiency stress of citrus leaf. The accuracies of SVM and ANN were 92.8% and 92.2%, respectively. These reported ML methods based on spectra or images can be used to distinguish the symptomatic trees seriously infected by *Ca*Las and the healthy ones, but cannot achieve early detection for HLB. Because the acquired information from spectra or images is not able to be directly linked to the changes in molecular indicators. The molecular indicators, including metabolites such as some hormones and antioxidants (some flavonoids), are the determiners of phenotypes or biological functions, which can reflect the degree of *Ca*Las infection and the physiological status of trees before the observable or photo-imageable symptoms appear.

ML algorithms present opportunities to recognize important metabolites through handling high-dimensional, multicollinear, and non-independent metabolomics data. Once combined, the power of metabolomics and ML could be taken to their extreme and even magnified perfectly. In this study, integrated with UHPLC/MS-based nontargeted metabolomics, six ML algorithms including regularized logistic regression (LR-L1 and LR-L2), random forest (RF), gradient-boosted decision tree (GBDT), support vector machines (SVM), and multilayer perception (MLP) were individually employed to build the classifier for the early diagnosis of HLB. It was finally demonstrated that those newly developed classifiers had different characteristics and prediction performances. Especially, the predictors based on LR-L2 and GBDT performed best for the datasets used in the current study. The methodologies developed in this study have not been proposed in any existing work to the best of our knowledge. Moreover, the significant differential metabolites as biomarkers yielded from the predictors were identified, and their content change and functions involved in metabolic pathways were also discussed.

**Figure 1:**
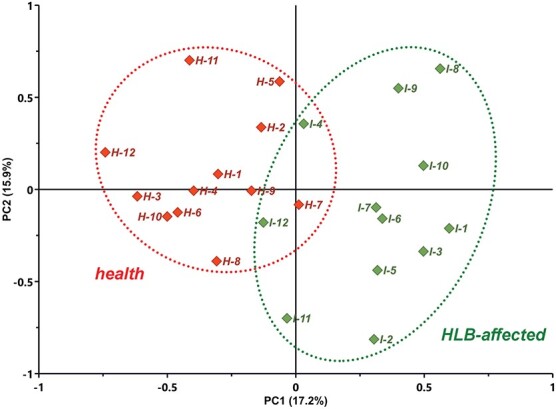
Score plot of principal component analysis (PCA) visualizing recognizable differences between citrus trees of healthy group (H-1 to H-12) and HLB-affected group (I-1 to I-12) in metabolic level.

## Results

### Metabolic variations of citrus trees at the early stage of HLB

At the time point of seven weeks after being infected by *Ca*Las, no phenotypic changes between the leaves from the infected trees and the healthy could be observed. The metabolic profiles of leaves between them were compared by PCA. Overall, as shown in Figure 1, the score plot displayed a recognizable distance between the sample clusters of the HLB-affected group from the healthy group, indicating the significant metabolic variations, which explained 33.1% of the total variation on the PC1 and PC2 axes. At the early stage of *Ca*Las infection, the trees exhibited a strong stress response to the heterologous grafts, but the events that took place in the infected trees and the healthy ones were vastly different. In the process of fighting the bacteria, the infected trees gradually developed a certain degree of homeostasis (but not tolerance) to the bacteria and showed stronger metabolic changes than the healthy trees. After a quick false alarm, the physiological status of healthy trees calmed down, meanwhile, their metabolic level went back to the previous normal. Except for that, it could be found that the distances between any two samples in each group were obvious, which implied the individual differences in the trees responded to the exposure. Thus, considering the great possibility of overfitting results from high-dimensional dataset with a feature size much larger than the sample size, it was hard to use the conventional multivariable statistical analysis methods, e.g. partial least squares-discriminant analysis (PLS-DA), to differentiate the two groups accurately and discover the metabolic differences between them, and the state-of-the-art ML algorithms would be better choices.

### Evaluation and comparison of machine learning models for early detection of HLB

Twelve-fold cross-validation was employed to measure and evaluate the performance and robustness of all the constructed ML models. The mean performance of six ML models for the test sets of four datasets (CN, CP, HN and HP) was shown in Table 1. Accuracy and F_1_ score were taken as the two main bases for judgment. From the results, it could be easily discovered that for most of the datasets, LR-L2 model and GBDT model outperformed for the prediction of citrus HLB among the six classifiers, and RF also performed quite well but only for HN dataset. As shown in Supplementary Figures S1 to S4, the AUCs for the six classifiers with different datasets were calculated. Most of the classifiers had AUCs equal to or close to 100%, which meant these classifiers were highly sensitive in terms of classification. To summarize, LR-L2 and GBDT were two robust predictors for the early detection of citrus HLB.

**Table 1:** Mean confusion matrices and performance indicators for evaluating six machine learning classifiers based on datasets of four acquisition sources for Huanglongbing detection

**Data source**	**Classifier**	**Confusion matrix**				**Accuracy (%)**	**Sensitivity (%)**	**Specificity (%)**	**Precision (%)**	**F**_**1**_ **Score (%)**	**MCC (%)**	**AUC (%)**
		**TN** ^**a**^	**FP**	**FN**	**TP**							
CN^**b**^	LR-L1	8	4	0	12	83.33 ± 23.57	100.00 ± 0.00	66.67 ± 47.14	83.33 ± 23.57	88.89 ± 15.71	66.67 ± 47.14	100.00 ± 0.00
	LR-L2	11	1	0	12	95.83 ± 13.82	100.00 ± 0.00	91.67 ± 27.64	95.83 ± 13.82	97.22 ± 9.21	91.67 ± 27.64	100.00 ± 0.00
	RF	8	4	0	12	83.33 ± 23.57	100.00 ± 0.00	66.67 ± 47.14	83.33 ± 23.57	88.89 ± 15.71	66.67 ± 47.14	100.00 ± 0.00
	GBDT	11	1	0	12	95.83 ± 13.82	100.00 ± 0.00	91.67 ± 27.64	95.83 ± 13.82	97.22 ± 9.21	91.67 ± 27.64	100.00 ± 0.00
	SVM	5	7	0	12	70.83 ± 24.65	100.00 ± 0.00	41.67 ± 49.30	70.83 ± 24.65	80.56 ± 16.43	41.67 ± 49.30	100.00 ± 0.00
	MLP	10	2	1	11	87.50 ± 21.65	91.67 ± 27.64	83.33 ± 37.27	n/a^**c**^	n/a	75.00 ± 43.30	100.00 ± 0.00
CP	LR-L1	8	4	0	12	83.33 ± 23.57	100.00 ± 0.00	66.67 ± 47.14	83.33 ± 23.57	88.89 ± 15.71	66.67 ± 47.14	91.67 ± 27.64
	LR-L2	11	1	0	12	95.83 ± 13.82	100.00 ± 0.00	91.67 ± 27.64	95.83 ± 13.82	97.22 ± 9.21	91.67 ± 27.64	100.00 ± 0.00
	RF	9	3	0	12	87.50 ± 21.65	100.00 ± 0.00	75.00 ± 43.30	87.50 ± 21.65	91.67 ± 14.43	75.00 ± 43.30	100.00 ± 0.00
	GBDT	11	1	0	12	95.83 ± 13.82	100.00 ± 0.00	91.67 ± 27.64	95.83 ± 13.82	97.22 ± 9.21	91.67 ± 27.64	100.00 ± 0.00
	SVM	6	6	0	12	75.00 ± 25.00	100.00 ± 0.00	50.00 ± 50.00	75.00 ± 25.00	83.33 ± 16.67	50.00 ± 50.00	100.00 ± 0.00
	MLP	7	5	0	12	79.17 ± 24.65	100.00 ± 0.00	58.33 ± 49.30	79.17 ± 24.65	86.11 ± 16.43	58.33 ± 49.30	91.67 ± 27.64
HN	LR-L1	7	5	0	12	79.17 ± 24.65	100.00 ± 0.00	58.33 ± 49.30	79.17 ± 24.65	86.11 ± 16.43	58.33 ± 49.30	100.00 ± 0.00
	LR-L2	8	4	0	12	83.33 ± 23.57	100.00 ± 0.00	66.67 ± 47.14	83.33 ± 23.57	88.89 ± 15.71	66.67 ± 47.14	100.00 ± 0.00
	RF	9	3	0	12	87.50 ± 21.65	100.00 ± 0.00	75.00 ± 43.30	87.50 ± 21.65	91.67 ± 14.43	75.00 ± 43.30	100.00 ± 0.00
	GBDT	11	1	0	12	95.83 ± 13.82	100.00 ± 0.00	91.67 ± 27.64	95.83 ± 13.82	97.22 ± 9.21	91.67 ± 27.64	95.83 ± 13.82
	SVM	6	6	0	12	75.00 ± 25.00	100.00 ± 0.00	50.00 ± 50.00	75.00 ± 25.00	83.33 ± 16.67	50.00 ± 50.00	100.00 ± 0.00
	MLP	8	4	1	11	79.17 ± 32.00	91.67 ± 27.64	66.67 ± 47.14	79.17 ± 32.00	n/a	58.33 ± 64.01	91.67 ± 27.64
HP	LR-L1	8	4	0	12	83.33 ± 23.57	100.00 ± 0.00	66.67 ± 47.14	83.33 ± 23.57	88.89 ± 15.71	66.67 ± 47.14	100.00 ± 0.00
	LR-L2	9	3	0	12	87.50 ± 21.65	100.00 ± 0.00	75.00 ± 43.30	87.50 ± 21.65	91.67 ± 14.43	75.00 ± 43.30	100.00 ± 0.00
	RF	5	7	0	12	70.83 ± 24.65	100.00 ± 0.00	41.67 ± 49.30	70.83 ± 24.65	80.56 ± 16.43	41.67 ± 49.30	100.00 ± 0.00
	GBDT	11	1	1	11	91.67 ± 18.63	91.67 ± 27.64	91.67 ± 27.64	n/a	n/a	83.33 ± 37.27	100.00 ± 0.00
	SVM	5	7	0	12	70.83 ± 24.65	100.00 ± 0.00	41.67 ± 49.30	70.83 ± 24.65	80.56 ± 16.43	41.67 ± 49.30	100.00 ± 0.00
	MLP	9	3	2	10	79.17 ± 32.00	83.33 ± 37.27	75.00 ± 43.30	n/a	n/a	58.33 ± 64.01	83.33 ± 37.27

a TN: true negative; TP: true positive; FN: false negative; FP: false positive.

b CN: C_18_ column–negative ion mode; CP: C_18_ column–positive ion mode; HN: HILIC column–negative ion mode; HP: HILIC column–positive ion mode.

c n/a: not applicable

### Confirmed LR-L2 and GBDT models with top-ranked and annotated features

**Figure 2:**
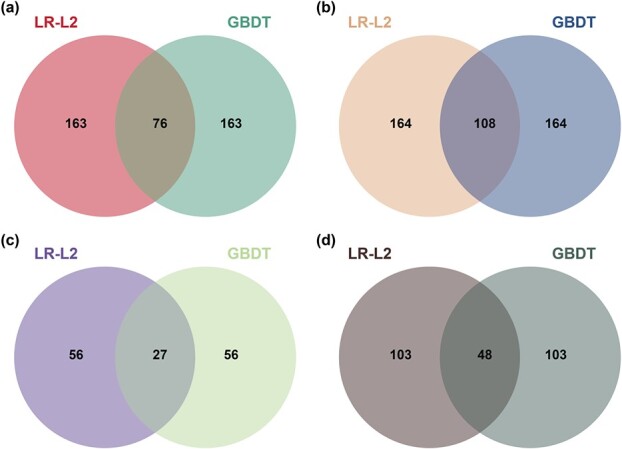
Counts of top-ranked metabolite features from LR-L2 and GBDT classifiers of four data sources. (a) C_18_ column–negative ion mode (CN). (b) C_18_ column–positive ion mode (CP). (c) HILIC column–negative ion mode (HN). (d) HILIC column–positive ion mode (HP)

As shown in Figure 2, there were 239, 272, 83 and 151 differential features resulting from the CN, CP, HN and HP datasets, respectively, which were the one-third of top-ranked features as the output of the LR-L2 classifier and GBDT classifier. Among them, 108, 161, 65 and 135 ones for CN, CP, HN and HP sources, respectively, were annotated by the systematic identification strategy. Then the datasets of those annotated features were extracted to re-perform LR-L2 classifiers and GBDT classifiers. The results of the performance evaluation and comparison were listed in Table 2. For the LR-L2 classifier, identical accuracies of 87.50% were acquired from the four datasets, the same is true of the F_1_ score of 91.67% and MCC of 75.00%. As for the GBDT classifier, the accuracies given out by the CN, CP, HN and HP datasets were 95.83%, 91.67%, 83.33% and 91.67, respectively. Except for the GBDT classifier based on the HN dataset, all the AUCs got from other classifiers were 100%. The confusion matrices of the LR-L2 classifier and the GBDT classifier were shown in Supplementary Figures S5 and S6, respectively. Compared with the classifiers based on all features, the overall prediction performance is decreased to some extent due to the reduction of feature size, but all the obtained classifiers were still robust with the least average accuracy greater than 83.33%. It was worth noting that as the confirmed predictors with all the built-in metabolites annotated, they would be more reliable in practice.

**Table 2:** Mean confusion matrices and performance indicators for evaluating two preferable machine learning classifiers based on datasets of top-ranked features from four acquisition sources for Huanglongbing detection

**Data source**	**Classifier**	**Confusion matrix**				**Accuracy (%)**	**Sensitivity (%)**	**Specificity (%)**	**Precision (%)**	**F**_**1**_ **score (%)**	**MCC (%)**	**AUC (%)**
		**TN** ^**a**^	**FP**	**FN**	**TP**							
CN^**b**^	LR-L2	9	3	0	12	87.50 ± 21.65	100.00 ± 0.00	75.00 ± 43.30	87.50 ± 21.65	91.67 ± 14.43	75.00 ± 43.30	100.00 ± 0.00
	GBDT	11	1	0	12	95.83 ± 13.82	100.00 ± 0.00	91.67 ± 27.64	95.83 ± 13.82	97.22 ± 9.21	91.67 ± 27.64	100.00 ± 0.00
CP	LR-L2	9	3	0	12	87.50 ± 21.65	100.00 ± 0.00	75.00 ± 43.30	87.50 ± 21.65	91.67 ± 14.43	75.00 ± 43.30	100.00 ± 0.00
	GBDT	10	2	0	12	91.67 ± 18.63	100.00 ± 0.00	83.33 ± 37.27	91.67 ± 18.63	94.44 ± 12.42	83.33 ± 37.27	100.00 ± 0.00
HN	LR-L2	9	3	0	12	87.50 ± 21.65	100.00 ± 0.00	75.00 ± 43.30	87.50 ± 21.65	91.67 ± 14.43	75.00 ± 43.30	100.00 ± 0.00
	GBDT	9	3	1	11	83.33 ± 23.57	91.67 ± 27.64	75.00 ± 43.30	n/a^**c**^	n/a	66.67 ± 47.14	95.83 ± 13.82
HP	LR-L2	9	3	0	12	87.50 ± 21.65	100.00 ± 0.00	75.00 ± 43.30	87.50 ± 21.65	91.67 ± 14.43	75.00 ± 43.30	100.00 ± 0.00
	GBDT	11	1	1	11	91.67 ± 18.63	91.67 ± 27.64	91.67 ± 27.64	n/a	n/a	83.33 ± 37.27	100.00 ± 0.00

a TN: true negative; TP: true positive; FN: false negative; FP: false positive.

b CN: C_18_ column–negative ion mode; CP: C_18_ column–positive ion mode; HN: HILIC column–negative ion mode; HP: HILIC column–positive ion mode.

c n/a: not applicable

### Discovered metabolic markers and pathways relevant to early stage of HLB

**Figure 3:**
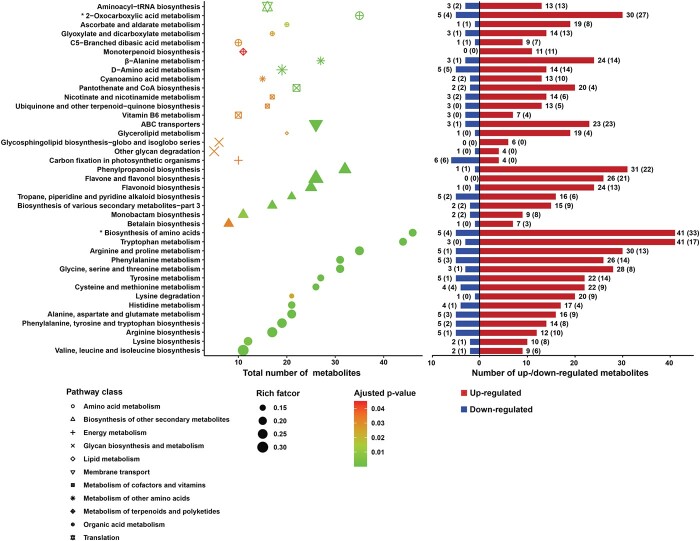
Bubble plot depicting results of metabolic pathway enrichment analysis enclosed with the number of up-regulated/down-regulated metabolites in each pathway. Asterisk indicates the global and overview maps. Rich factor is the ratio of metabolite numbers enriched in the pathway term to all metabolite numbers annotated in that pathway term. The greater the rich factor, the greater the degree of pathway enrichment. Adjusted p-value is the corrected p-value by false discovery rate (FDR), and a lower value indicates a greater pathway enrichment. The figures out of brackets represent the number of metabolites in a pathway, including metabolites related to the enzymes within a pathway, even if they are not in the original definition of the KEGG pathways; the figures in brackets represent the number of metabolites directly included in a pathway.

After combination and deduplication, a total of 331 differential metabolites screened out from LR-L2 and GBDT classifiers (Supplementary Data S2) were turned to metabolic pathway enrichment analysis. As a result, 39 different metabolic pathways with statistical significance were mapped, including 25 very significant ones with adjusted p-value less than 0.01, and 14 significant ones with adjusted p-value less than 0.05. The results of pathway enrichment analysis and the number of upregulated/downregulated differential metabolites in each pathway are shown in Figure 3. Among these pathways, eight were involved in amino acid metabolism (primary amino acids and other amino acids), seven were involved in biosynthesis of various secondary metabolites (flavonoid, phenylpropanoid, alkaloid, monobactam, betalain), four were involved in organic acid metabolism, four were involved in metabolism of cofactors and vitamins, two were involved in glycan biosynthesis and metabolism (glycosphingolipid and other glycans), one was involved in energy metabolism (carbon fixation in photosynthetic organisms), one was involved in lipid metabolism (glycerolipid), one was involved in membrane transport (ABC transporters), and one was involved in translation (aminoacyl-tRNA biosynthesis). Overall, most of the metabolites in the pathway of carbon fixation in photosynthetic organisms were downregulated, and those distributed in other pathways were upregulated (see Figure 2 and Supplementary Data S3). This result is quite consistent with the previous reports [19, 39–45].

**Figure 4:**
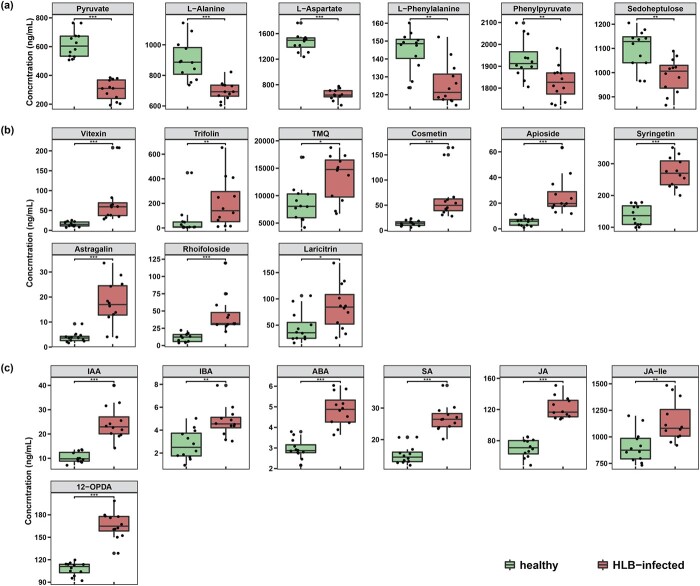
Content change of some representative metabolites in three significant metabolic pathways detected from leaves of HLB-affected trees compared to healthy trees. (a) Carbon fixation in photosynthetic organisms. (b) Flavone and flavonol biosynthesis. (c) Plant hormone signal transduction. TMQ: 3,7,4′-Tri-O-methylquercetin; IAA: Indole-3-acetic acid; IBA: Indole-3-butyric acid; ABA: Abscisic acid; SA: Salicylic acid; JA: (−)-Jasmonic acid; JA-Ile: (−)-Jasmonic acid-L-Isoleucine; 12-OPDA: 12-Oxo-10,15(Z)-phytodienoic acid. Asterisks indicate significance of difference by Students’ t-test: ^*^ p-value <0.05; ^**^ p-value <0.01; ^***^ p-value <0.001.

As shown in Figure 4a, six metabolites located on the pathway of carbon fixation in photosynthetic organisms, including pyruvate, L-alanine, L-aspartate, L-phenylalanine, phenylpyruvate and sedoheptulose were detected to be significantly downregulated in the infected group compared to the healthy group. Peng et al. [39] reported that the leaves of “Chongyi” wild mandarin (*Citrus reticulata*) showed downregulation of transcripts and metabolites relevant to carbon fixation after 18 weeks of graft-inoculation with *Ca*Las-infected buds by the integrated transcriptomics and metabolomics analyses. Wei et al. [40] also reported that the number of differentially expressed genes (DEGs) related to photosynthesis detected in “Valencia” orange (*C. sinensis*) at one and five days of post-*Ca*Las-infected psyllid infection was greatly less than that of healthy psyllid infection. Suh et al.’s [19] research indicated the obvious downregulation of metabolites involved in carbon fixation in photosynthetic organisms in the leaves of four-year-old “Hamlin” orange (*C. sinensis*) trees naturally infected by *Ca*Las before experiments.

Amino acids are the basic material source for plants to synthesize various important secondary metabolites. A total of 128 amino acids (or amino acid derivatives) were detected to be markedly upregulated in the infected group compared with healthy plants (Supplementary Data S1), which agreed with Killiny et al.’s [41] and Hung et al.’s [42] discoveries.

Flavonoids are naturally existing antioxidants in plants, some of which play the roles of defense substances against extrinsic pathogens. It was demonstrated that suppressing the reactive oxygen species using antioxidants such as flavonoids would be a hopeful approach to control HLB [43]. Hijaz et al. [44] found that some structurally related hydroxycinnamate compounds including flavonoid glycosides and polymethoxylated flavones increased up to ten folds in leaves from “Hamlin” and “Valencia” sweet orange trees in response to *Ca*Las infection. Hung et al. [42] found that *Ca*Las infection could increase the accumulation of the majority of the flavonoids in “Hamlin” oranges and “Marcotte” mandarins. Xue et al. [45] also reported that the expression of some genes involved in the phenylpropanoid branching pathway (4CL, HCT, CHI, CHS, CYP, and C12R) was increased in asymptomatic and early leaves of HLB affection, and decreased in late leaves. In the present study, nine flavonoid compounds, including vitexin, trifolin, 3,7,4′-tri-O-methylquercetin (TMQ), cosmetin, apioside, syringetin, astragalin, rhoifoloside, laricitrin, were discovered to be significantly upregulated in the infected group compared to the healthy plants (Figure 4b), which gave full support to the previous report.

Moreover, it is well-known that phytohormones are important chemical substances for plant stress response. Peng et al. [39] reported that DEGs related to SA were mainly detected in the *Ca*Las-infected leaves, and all those related to JA were upregulated regardless of tissue type. Wei et al. [40] also reported the upregulation of DEGs involved in phytohormone. In our study, as shown in Figure 4c, seven metabolites involved to plant hormone signal transduction were detected to be significantly upregulated, including indole-3-acetic acid (IAA), indole-3-butyric acid (IBA), abscisic acid (ABA), salicylic acid (SA), (−)-jasmonic acid (JA), (−)-jasmonic acid-L-Isoleucine (JA-Ile) and 12-oxo-10,15-(Z)-phytodienoic acid (12-OPDA). It is speculated that in the early stage of HLB, infected plants may activate a non-uniform and lagged enhancement of defense responses to *Ca*Las at the expense of photosynthesis, contributing to elevated metabolic levels for survival and tolerance.

## Discussion

### Machine learning methods outperforming on HLB prediction

In the field of horticulture research, ML is mainly applied to recognize and predict crop disease and stress behaviour [46]. Efficient and accurate prediction of plant diseases is a prerequisite for plant protection management. As the significance of precision agriculture, the prediction and quantification of plant diseases will be more important than identification and classification. Conducting such studies can prevent crop diseases at an early stage and reduce pesticide costs. In the current study, specialized ML predictors were developed for the identification of asymptomatic HLB-affected citrus trees by scrutinizing the metabolites in leaves. Owing to the lack of dataset for HLB of early stage, we created the datasets by the UHPLC/MS-based metabolomics method. Six supervised ML classifiers including LR-L1, LR-L2, RF, GBDT, SVM and MLP were trained to distinguish the healthy plants from the infected ones. Among these classifiers, the LR-L2 and the GBDT achieved the highest average predictive accuracy of up to 95.83% by means of the analysis of the metabolites feature sets.

The HLB always occurs quietly, and the severity of after affection also changes all the time as the citrus trees grow, which leads to difficulty in conducting credible early identification. Based on the developed strategy in this study, sensitive and accurate methods could be established for the prediction of HLB at the early stage. Automated disease prediction can be achieved by combining machine learning models and domain knowledge of HLB. Image processing and recognition techniques could reportedly help with identifying plant diseases. By the convolutional neural networks (CNN) algorithm, it could yield a prediction accuracy rate greater than 90% [47, 48]. Thus far, a few research have already been carried out to predict the onset of HLB development. The techniques such as image classification/segmentation were used for HLB identification [49]. Various algorithms were applied to categorize healthy and infected plants or give out the probability of HLB affection. By using the artificial neural network (ANN) model, with plenty of data, the highest classification accuracy was 98.5% [49]. Although these image recognition-based methods have been practiced in the field of early prediction of HLB for several years, different lighting conditions impacting the prediction performance were later found to be a potential obstacle [50]. Physiological evaluations based on the more susceptible features like levels of nutrition elements, phytopigments and photosynthesis indexes, and metabolites can help to tackle this challenge. The excellent applicability for predicting HLB at the early stage by the metabolite features was verified by the present study.

In terms of performance, all classifiers built in this study performed well. LR-L2 and GBDT were selected as the best performing classifiers, probably because both two classifiers used the regularization technique to improve the performance and included cross-validation to result in the optimal thresholds and parameters. SVM had the lowest performance among others. It could be that the support vector was mainly decided by the features sited at the cluster margin. If the slightly larger overlap between the sample distribution within two clusters was existing, the classification performance of SVM would decrease apparently. Various ML methods had different prediction accuracies. Therefore, it is necessary to try a variety of ML methods and determine the most suitable method to use. Experience in this area also needs to be accumulated in practice. It was also noted that the relatively small sample size with a large feature size of the dataset was a limitation for the ML modeling.

The differences between ML and the conventional statistical methods on feature selection were compared. For the datasets used in this study, after filtering with p-value <0.05, only 257, 272, 83 and 113 features were left for the datasets of CN, CP, HN and HP, respectively. And if filtering with |Log2 Fold Change| ≥ 1 (FC, ratio of medians of two groups), only 137, 128, 25 and 28 features were retained, respectively. Probably due to being extremely stringent and acting as “one size fits all”, these two conventional filter criteria resulted in too few features. In contrast, ML methods were more flexible on feature filtering. They calculated the contribution size of each feature to the model (e.g. coefficients of independent variables in LR-L2, which represented the importance of individual features), and considered the pattern of all features to make the final classification decision. Also, they could automatically optimize the parameters through cross-validation, selected the best threshold by the point that the TPR and the FPR crossed in the ROC curve, determined the optimal model, and applied it to rank the features by their importance.

### Combination of UHPLC/MS-based nontargeted metabolomics and machine learning boosting early accurate identification of HLB

Bacteria diseases of the higher plant can be detected through direct or indirect strategies. For citrus HLB, the direct strategies consist of conventional PCR, qPCR and chemical staining. They detect amounts of *Ca*Las via amplifying marker genes, or microscopically inspect secretions of psyllids using chemical staining, by which whether the trees are diseased can be concluded. Recently, it was proved by the practice that the qPCR method was more robust for symptomatic plants [9]. But for the asymptomatic plants at the early stage of HLB affection, it was demonstrated ineffective. As shown in Supplementary Data S2, among the twelve leaf samples collected at the timepoint of seven weeks after *Ca*Las infection, only one sample (I-9) could be correctly determined to be HLB-affected by two independent qPCR tests. The sole successful practice of the Coomassie Brilliant Blue Staining was taken place on the five-month-old seedlings of *Ca*Las-infected Valencia sweet orange trees with young flush [10].

The indirect strategies estimate the healthy status of plants by measuring and comparing physiological (e.g. chlorophyll fluorescence index) and morphological variations (e.g. canopy size and density, leaf area and weight) or differences in chemicals released (e.g. hormones, antioxidants) in the infected and healthy groups during defense responses. These diverse plant-derived changes can be efficiently measured by a great variety of analytical techniques, such as UHPLC/MS, and the data in high-dimensional form can be parsed by powerful ML algorithms [46], which enable the accurate detection of HLB. The nontargeted metabolomics can conduct unbiased and accurate detection for almost all metabolites in diseased plants and uncover their content changes. In the present investigation, the power of ML and metabolomics were united and magnified perfectly. By combining the UHPLC/MS-based nontargeted metabolomics strategy, two predictors (LR-L2 and GBDT) among six ones were discovered to be outperformed with an average prediction accuracy above 95% for early HLB detection. For the GBDT model, the mean specificities were 91.67% for all datasets. But for the other five ML models, including LR-L2, the mean specificities were relatively lower than those of GBDT, especially for the HN dataset and HP dataset. In this regard, the GBDT model was much more practical. The other five models might exhibit a high probability to classify the healthy plants as the infected plants, due to they were over-sensitivity to predicting HLB affection. Considering the detection method, four different column–ion mode combinations, i.e. CN, CP, HN and HP were compared. It was concluded that CN and CP were the better methods for data acquisition than HN and HP, the predictors built by which worked better than others. It is possible that by using these two combinations, more metabolites could be detected, and more accurate and repeatable peak areas could be obtained (Table 1). Some significant metabolite features involved in multiple metabolic pathways were screened out based on those two ML predictors. It was noticed that various ML algorithms have different tendentiousness in terms of the ranking of important features. Many significant metabolites yielded from LR-L2 and GBDT algorithms were different (Figure 1). In this case, the united application of multiple ML methods would improve the reliability of the result. Additionally, the practicability of those predictors and the effectiveness of those discovered biomarkers were verified by the metabolic pathway analysis and content change analysis, which were also completely supported by the previous publications.

The relevant agricultural communities can freely use the predictors developed in the current research to perform the early detection of HLB. Any one of the four predictors (CN/CP/HN/HP) based on the GBDT classifier or the LR-L2 classifier can be applied individually or combinedly according to the practical conditions. Those metabolic biomarkers included in the predictors (Supplementary Data S3) should be taken as the targeted compounds and quantitatively detected by any available LC–MS instrument methods, such as the selected ion monitoring (SIM) or multiple reaction monitoring (MRM), to acquire the test dataset. In the coming future, it is also hopeful that the current strategy could work together with UAV photographing to achieve high-throughput and accurate early identification of HLB-affected plants in the fields. The images captured utilizing a multispectral or hyperspectral camera on a UAV system would be firstly implemented to perform the pre-detection via the reported imaging-based ML predictors [11], by which the ranges of the plants questionably infected could be determined. Then the ML predictors developed in the current study would be applied after the targeted sampling and targeted metabolites quantification, to finally reach diagnostic conclusions with perfect confidence.

## Materials and methods

### Chemicals and reagents

The authentic internal standards, including L-carnitine-d_3_, salicylic acid-d_6_, D-sorbitol-^13^C_6_ and L-proline-2,5,5-d_3_ were ordered from Sigma-Aldrich (St. Louis, MO, USA). Water, acetonitrile, methanol, and formic acid (all UHPLC–MS grade) were purchased from Fisher Scientific (Fair Lawn, NJ, USA).

### Plant materials

‘Midsweet’ sweet orange (*C. sinensis* (L.) Osbeck) trees were planted and raised in the greenhouse at Citrus Research and Education Center of University of Florida (Lake Alfred, FL, USA). The budwoods of ‘Midsweet’ were grafted onto US-802 [*C. grandis* (L.) Osbeck (‘Siamese’ pummelo) × *P. trifoliata* (‘Gotha Road’)] rootstock. Exactly 12 months after grafting, we inoculated the seedlings with scions from completely pathogen-free and seriously *Ca*Las-infected sour oranges, yielding both healthy and *Ca*Las-infected trees, respectively. The certified pathogen-free material was provided by the Division of Plant Industry, Florida Department of Agriculture & Consumer Services. As the *Ca*Las-infected source materials used for grafting, the suitable budwoods were cut from the trees cultured in the field and tested using the leaves on those branches for confirmation of HLB affection by qPCR referring to the reported method [24]. All the grafting work was performed by a professional grafter. Seven weeks post-exposure, we collected 10 to 14 leaves at random from each of twelve individual healthy and infected trees (biological replicate, *n* = 12) on August 17, 2018. We froze the collected samples in liquid nitrogen right away and then stored them at −80°C. The health/disease status of all the leaf samples was tested by qPCR in two different labs (Supplementary Data S2).

### Sample preparation for UHPLC/MS analysis

Leaf samples were ground into fine powder under the protection of liquid nitrogen. Sixty-milligram samples were weighed and put to 1.5 mL Eppendorf tubes, and mixed with 1 mL cooled 80% methanol aqueous solutions, 20 μL salicylic acid-d_6_, 20 μL L-carnitine-d_3_, 20 μL L-proline-2,5,5-d_3_ and 20 μL D-sorbitol-^13^C_6_ as internal standards. The individual concentration of the internal standard solution was 10 ppm. The mixture was vortexed for 10 min, sonicated in an ice-cold ultrasonic bath for 30 min, and then centrifuged at 20000 rpm for 10 min at 4°C. The supernatant was collected and transferred to a new tube, then filtered using a 0.22 μm nylon membrane filter (Thermo Fisher Scientific, Waltham, MA, USA). We prepared the quality control (QC) sample through pooling isometric extract solutions of all samples.

### UHPLC/MS analysis

Chromatographic separation was carried out on a Vanquish Flex Binary UHPLC (Thermo Fisher Scientific, Waltham, MA, USA) with a Waters Acquity CSH C_18_ column (150 × 2.1 mm, 1.7 μm) and an Agilent InfinityLab Poroshell 120 HILIC-Z (150 × 2.1 mm, 2.7 μm) column, respectively. The column temperature was held at 40°C. For the reversed-phase (RP) C_18_ separation system, the mobile phase was composed of water with 0.1% formic acid (phase A) and acetonitrile with 0.1% formic acid (phase B), and the gradient elution program was: 0–22min, 2–38% B; 22–23 min, 38–42% B; 23–28 min, 42–70% B; 28–29 min, 70–100% B; 29–30 min, 100% B. The column was re-equilibrated with the initial mobile phase ratio for 6 min. For the hydrophilic interaction liquid chromatography (HILIC) separation system, the mobile phase was composed of 10 mM ammonium acetate (pH 9.0) in water with 0.25 mM methylphosphonic acid (phase A) and 10 mM ammonium acetate (pH 9.0) / acetonitrile (10/90) (phase B), and the elution program was: 0–2 min, 90% B; 2–8 min, 90–70% B; 8–10 min, 70–40% B; 10–18 min, 40% B. The column was re-equilibrated with the initial mobile phase ratio for 9 min. The flow rate was maintained at 0.3 mL/min. The injection volume was at 3 μL.

A Q Exactive Plus UHPLC/mass spectrometer system (Thermo Fisher Scientific, Waltham, MA, USA) was employed to perform the detection. The parameters for the HESI source were set based on experience as follows: capillary temperature, 275°C; heater temperature, 300°C; sheath gas flow, 50 arb; auxiliary gas flow, 10 arb; purge gas flow, 0 arb/2 arb (C_18_/HILIC); spray voltage, 3.5 kV/3 kV (positive/negative); S-lens RF level, 55%. The mass spectrometer adopted the Full-MS/ddMS^2^ scan in positive and negative ion modes, respectively. Mass spectra were obtained in the m/z range of 70 to 1050, and the resolution was set to 70 000. The automatic gain control (AGC) was 3 × 10^6^ and the injection time (IT) was 100 ms. For MS/MS scans, step-normalized collision energy was set to 20, 40, and 60 V with a resolution of 17 500. AGC is 1 × 10^5^ and IT is 50 ms. A data-dependent analysis scan was applied to trigger the second-stage fragmentation, whereby the 12 most intense precursor ions at each scan point of the MS were selected as target precursor ions for further MS/MS fragmentation. The dynamic exclusion function was turned on to evade repetitive analyses.

### Data pre-processing and feature selection

The Compound Discoverer v3.1 (Thermo Fisher Scientific, Waltham, MA, USA) was used to pre-process the data by the empirical workflow involving the nodes retention time aligning, peak detection and grouping, gap filling, peak area correction, molecular formula deduction, and database search. After the data pre-processing was finished, the results of internal standards and QC were first checked to validate the stability of the sample preparation process and instrument method. Only the metabolites with relative standard deviation (RSD%) of peak areas in QC samples less than 30% were kept in the downstream study. Then four feature tables derived from four different acquisition methods were exported, respectively, which were datasets of the combinations of one of two separate systems (reversed phase C_18_/HILIC) and one of two ion modes (positive/negative). The feature tables contained the retention time and the corresponding peak areas for all the detected features.

**Figure 5:**
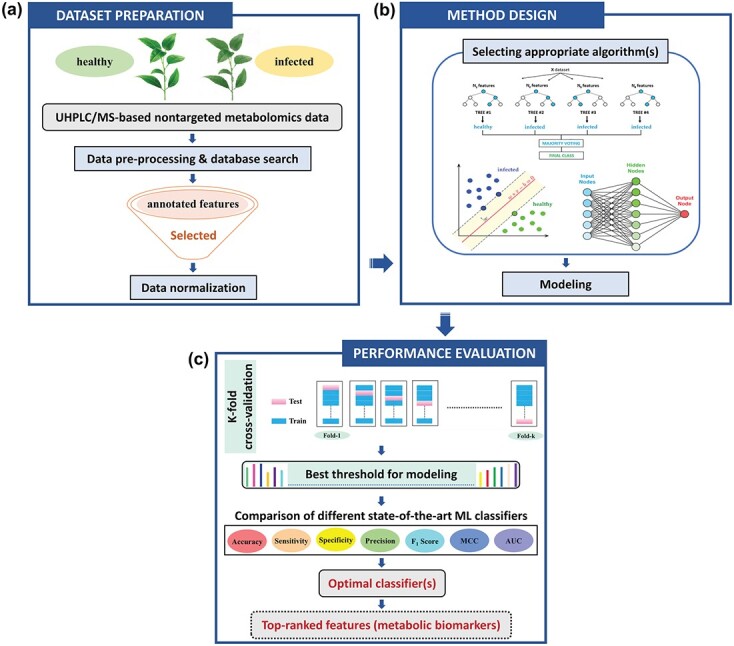
Schematic workflow of multiple machine learning (ML) modeling combined with UHPLC/MS-based nontargeted metabolomics for early accurate detection of Huanglongbing (HLB): (a) Dataset creation and feature selection; (b) Algorithms selection and ML modeling; (c) Model testing, optimization and implementation.

Feature selection was designed to improve the practicability of models and the interpretability of the discovered differential features (metabolites). All the annotated features were selected, and the ones without annotation were abandoned. Each dataset derived from C_18_ column–negative ion mode (CN), C_18_ column–positive ion mode (CP), HILIC column–negative ion mode (HN) and HILIC column–positive ion mode (HP) included twelve HLB-affected samples and twelve healthy samples, and each sample had 707, 816, 249 and 453 features, respectively. Considering too many features will be removed after pre-filtering by conventional criteria, e.g. p-value, the original feature size was kept for ML modeling. Then the data were scaled using the Z-score normalization by features in RStudio v2021.9.0.351 (RStudio, Inc., Boston, MA) [25] based on the R v4.1.0 platform (R Foundation for Statistical Computing, Vienna, Austria) [26]. The PCA was performed to check the original distribution of data after merging the four datasets and to visualize the metabolic variations of the samples within the HLB-affected group and the healthy group, which was completed by SIMCA v17. 0 (Sartorius Stedim Data Analytics AB, Umeå, Sweden).

### Computational method and machine learning modeling

The pipeline for multiple ML modeling combined with nontargeted metabolomics for the early detection of HLB is schematically shown in Figure 5. Data organization was the initial step. The CN, CP, HN and HP datasets consisting of the twelve healthy samples and an equal number of HLB-affected samples were organized for ML modeling. A variety of ML classifiers could be adopted. Considering the data feature including continuity and high dimensionality, and to ensure the universality and accessibility of the developed models, after the data pre-processing and organization steps, six supervised ML classifiers including LR-L1 and LR-L2 [27], RF [28], GBDT [29], SVM [29] and MLP [30] were selected and employed. The standard procedure for evaluating classifier performance was k-fold cross-validation [31]. Considering the sample size, twelve-fold cross-validation was applied. Throughout this process, without overlap, the dataset was randomly divided into twelve subsets. Then, the eleven servings were used as the training dataset and the rest were taken as the test set for model evaluation. This process was repeated twelve times to allow each subset to be used as a test set exactly once. Finally, we calculated the average performance over all the twelve test sets. Important performance evaluation measures for early HLB prediction were discussed in detail in the following subsections. All these metrics were based on the four basic elements that composed the confusion matrix: true negatives (TN) represent the number of healthy plants classified correctly, true positives (TP) represent the number of infected plants classified correctly, false negatives (FN) represent the number of infected plants classified as healthy incorrectly, and false positives (FP) represent the number of healthy plants classified as infected incorrectly.

All the ML modeling procedures for LR-L1, LR-L2, RF, GBDT, SVM and MLP were run through the integrated development environment (IDE) Spyder (Anaconda 3) v4.2.5 (Spyder Project Contributors) based on Python v3.8.8 (Python Software Foundation) [32]. The parameters of the models were optimized by internal cross-validation of training data. It was completed by grid-searching a range of values and picking the thresholds of true-positive rate (TPR) and false-positive rate (FPR) that generate the best region under the receiver operating characteristic (ROC) curve.

### Model evaluation

TP, FP, TN and FN were the evaluation parameters used to assess ML classifiers. The performance of classification was assessed by its accuracy, specificity, sensitivity, precision, recall, and F_1_ score. For two-class problems, all of the performance criteria in this work were illustrated as follows:

Accuracy.

Accuracy is the ratio of the number of samples categorized correctly over the complete number of samples [33]:

(1) \begin{equation*} \mathrm{Accuracy}=\frac{TP+ TN}{TP+ FP+ TN+ FN} \end{equation*}

Sensitivity (recall) and specificity.

Sensitivity (also called recall, or TPR) is the ratio of the number of accurately classified positive samples over the total positive samples [33]:

(2) \begin{equation*} \mathrm{Sensitivity}=\frac{TP}{TP+ FN} \end{equation*}

whereas the specificity is expressed as the ratio of the number of accurately grouped negative samples to the full number of negative samples [33]:

(3) \begin{equation*} \mathrm{Specificity}=\frac{TN}{TN+ FP} \end{equation*}

Precision.

Precision (referred to as positive prediction value, PPV) is the ratio of the number of accurately classified positive samples to the complete number of samples predicted to be positive [33]:

(4) \begin{align*} \mathrm{Precision}=\frac{TP}{TP+ FP} \end{align*}

F-measure.

F-measure demonstrates the harmonic means of sensitivity and precision. F_1_ score is used as a single measure of performance of the test for the positive class [33]:

(5) \begin{equation*} {\mathrm{F}}_1\ \mathrm{score}=2\times \frac{Sensitivity\times Precision}{Sensitivity+ Precision}=\frac{2 TP}{2 TP+ FP+ FN} \end{equation*}

Matthews correlation coefficient (MCC).

MCC exemplifies the correlation between true and predicted labels [34]:

(6) \begin{equation*} \mathrm{MCC}=\frac{TP\times TN- FP\times FN}{\sqrt{\left( TP+ TN\right)\times \left( TP+ FN\right)\times \left( TN+ FP\right)\times \left( TN+ FN\right)}} \end{equation*}

Area under the curve (AUC).

ROC has been adopted in the ML community for the past few years to visualize and assess the trade-off between TPR and FPR [33]. To compare the results of classifiers, ROC can be calculated to a single scalar value called the area under the curve (AUC), which is experiencedly defined as a measure of classification quality [35]. AUC is not affected by the random choice of specific classification inception, so it is considered an important evaluation metric.

### Differential metabolites selection and identification

The one-third of top-ranked features yielded from the ML models with robust prediction performance from the datasets of four acquisition sources were empirically selected and merged as the differential metabolites. The rank numbers represented the importance of each feature in the corresponding model. The identification of differential metabolites, carried out by a systematic identification strategy, was based on the high-resolution mass spectrum (HRMS) via Compound Discoverer v3.1. The workflow begins with the use of the formula predictor to automatically infer the elemental composition of each characteristic peak from the exact mass of the protonated molecular ion, and their respective isobaric molecular ions. Maximum mass accuracy error is limited to ±3 ppm. The formulas were then submitted to recognize the known compounds, which were matched by the in-house KEGG pathway metabolite structure library. All the annotated metabolites were manually confirmed by their MS/MS fragmentation patterns, as well as other available physicochemical properties such as the retention time of authentic standard and cLogP values [36].

### Metabolic pathway enrichment analysis

The metabolic pathway enrichment analysis of differential metabolites was performed using “FELLA” package v1.12.0 [37] in RStudio v2021.9.0.351. The up-to-date data were retrieved from the Kyoto Encyclopaedia of Genes and Genomes (KEGG) database (Kanehisa Laboratories, Release 100.0, http://genome.jp/kegg) [38], with an organism-specific code “cit” for “Valencia” orange (*Citrus sineses*). To ensure completeness, the hypergeometric test is included in the function “runHypergeom” within the “FELLA” package. As in several over-representation analysis (ORA) implementations, the hypergeometric distribution is used to assess whether a biological pathway contains more hits in the input list than that would be expected given the chance of its size. Pathways are ranked according to their p-values after multiple-testing correction. It is worth noting that the results of this test will be different from the hypergeometric test using the original KEGG pathway, because the metabolite-pathway links are inferred from the KEGG maps. Those metabolites would be involved in a pathway if they can be reached from a metabolite in the upward-pointing pathways. Therefore, metabolites associated with enzymes in a pathway would be included in this pathway even though they do not exist in the original pattern of the KEGG pathway [37].

## Supplementary Material

Web_Material_uhac145

## Data Availability

The datasets and script of Python codes used for multiple ML modeling and external test refer to the GitHub website (https://github.com/Yu-Wang-Lab/Multiple_ML_modeling_for_HLB_prediction).

## References

[1] Graca JV . Citrus greening disease. *Annu Rev Phytopathol*. 1991;29:109–36.

[2] Jagoueix S , Bove JM, Garnier M. The phloem-limited bacterium of greening disease of citrus is a member of the alpha subdivision of the Proteobacteria. *Int J Syst Bacteriol*. 1994;44:379–86. 7520729 10.1099/00207713-44-3-379

[3] Texeira DC , Ayres J, Kitajima EW et al. First report of a Huanglongbing-like disease of citrus in Sao Paulo state, Brazil and association of a new Liberibacter species, “*Candidatus* Liberibacter americanus”, with the disease. *Plant Dis*. 2005;89:107–7. 10.1094/PD-89-0107A 30795297

[4] Kramer J , Simnitt S & Calvin L. Fruit and Tree Nuts Outlook: March 2020. 2020. https://www.ers.usda.gov/publications/pub-details/?pubid=98169.

[5] USDA . Citrus: world markets and trade. 2020. https://www.fas.usda.gov/data/citrus-world-markets-and-trade.

[6] Bové JM . Huanglongbing: a destructive, newly-emerging, century-old disease of citrus. *J Plant Pathol*. 2006;88:7–37.

[7] Tsai JH , Liu YH. Biology of *Diaphorina citri* (Homoptera: Psyllidae) on four host plants. *J Econ Entomol*. 2000;93:1721–5. 11142304 10.1603/0022-0493-93.6.1721

[8] Roistacher CN . (eds) Graft-Transmissible Diseases of Citrus: Handbook for Detection and Diagnosis (Food and Agriculture Organization of the United Nations, 1991).

[9] Li WB , Hartung JS, Levy L. Quantitative real-time PCR for detection and identification of *Candidatus* Liberibacter species associated with citrus huanglongbing. *J Microbiol Methods*. 2006;66:104–15. 16414133 10.1016/j.mimet.2005.10.018

[10] Pandey SS , Wang N. Targeted early detection of citrus Huanglongbing causal agent '*Candidatus* Liberibacter asiaticus' before symptom expression. *Phytopathology*. 2019;109:952–9. 30667340 10.1094/PHYTO-11-18-0432-R

[11] Lan YB , Huang ZX, Deng XL et al. Comparison of machine learning methods for citrus greening detection on UAV multispectral images. *Comput Electron Agric*. 2020;171:105234.

[12] Sankaran S , Ehsani R, Etxeberria E. Mid-infrared spectroscopy for detection of Huanglongbing (greening) in citrus leaves. *Talanta*. 2010;83:574–81. 21111177 10.1016/j.talanta.2010.10.008

[13] Sankaran S , Mishra A, Maja JM et al. Visible-near infrared spectroscopy for detection of Huanglongbing in citrus orchards. *Comput Electron Agric*. 2011;77:127–34.

[14] Wetterich CB , De Oliveira F, Neves R et al. Detection of Huanglongbing in Florida using fluorescence imaging spectroscopy and machine-learning methods. *Appl Opt*. 2016;56:15–23.

[15] Sanchez L , Pant S, Mandadi K et al. Raman spectroscopy vs quantitative polymerase chain reaction in early stage Huanglongbing diagnostics. *Sci Rep*. 2020;10:10101. 32572139 10.1038/s41598-020-67148-6 PMC7308309

[16] Yao MY , Fu GR, Xu J et al. In situ diagnosis of mature HLB-asymptomatic citrus fruits by laser-induced breakdown spectroscopy. *Appl Opt*. 2021;60:5846–53. 34263804 10.1364/AO.427856

[17] Albrecht U , Fiehn O, Bowman KD. Metabolic variations in different citrus rootstock cultivars associated with different responses to Huanglongbing. *Physiologie végétale*. 2016;107:33–44. 10.1016/j.plaphy.2016.05.030 27236226

[18] Fiehn O . Metabolomics—the link between genotypes and phenotypes. *Plant Mol Biol*. 2002;48:155–71. 11860207

[19] Suh JH , Guha A, Wang ZX et al. Metabolomic analysis elucidates how shade conditions ameliorate the deleterious effects of greening (Huanglongbing) disease in citrus. *Plant J*. 2021;108:1798–814. 34687249 10.1111/tpj.15546

[20] Cajka T , Fiehn O. Toward merging untargeted and targeted methods in mass spectrometry-based metabolomics and lipidomics. *Anal Chem*. 2016;88:524–45. 26637011 10.1021/acs.analchem.5b04491

[21] Raftery D , ed. Mass Spectrometry in Metabolomics. Springer: New York; 2014.

[22] Perez De Souza L , Alseekh S, Naake T et al. Mass spectrometry-based untargeted plant metabolomics. *Curr Protoc*. *Plant Biol*. 2019;4:e20100. 10.1002/cppb.20100 31743625

[23] Bzdok D , Altman N, Krzywinski M. Statistics versus machine learning. *Nat Methods*. 2018;15:233–4. 30100822 10.1038/nmeth.4642 PMC6082636

[24] Ananthakrishnan G , Choudhary N, Roy A et al. Development of primers and probes for genus and species specific detection of ‘*Candidatus* Liberibacter species’ by real-time PCR. *Plant Dis*. 2013;97:1235–43. 30722431 10.1094/PDIS-12-12-1174-RE

[25] Rstudio Team . RStudio: integrated development environment for R. 2021. http://www.rstudio.com.

[26] R Core Team . R: A Language and Environment for Statistical Computing. 2020. http://www.r-project.org/index.html.

[27] Qin J , Lou YF. L1–2 Regularized Logistic Regression, in Proceedings of *the 53rd Asilomar Conference on Signals, Systems, and Computers* 779–783. United States: Pacific Grove; 2019.

[28] Basu S , Kumbier K, Brown JB et al. Iterative random forests to discover predictive and stable high-order interactions. *P Natl A Sci*. 2018;115:1943–8. 10.1073/pnas.1711236115 PMC5828575 29351989

[29] Friedman JH . Stochastic gradient boosting. *Comput Stat Data Anal*. 2002;38:367–78.

[30] Shan J , Wang YY, Gao W. Prediction of chemical exergy of organic substances using artificial neural network-multi layer perceptron. *Energy Sources, Part A: Recovery, Utilization, and Environmental Effects*. 2018;40:1826–32.

[31] Krstajic D , Buturovic LJ, Leahy DE et al. Cross-validation pitfalls when selecting and assessing regression and classification models. *J Cheminformatics*. 2014;6:10. 10.1186/1758-2946-6-10 PMC3994246 24678909

[32] Van Rossum G , Drake FL Jr, eds. Python Tutoria Centrum voor Wiskunde en Informatica. Centrum voor Wiskunde en Informatica Amsterdam: Amsterdam; 1995.

[33] Sokolova M , Japkowicz N, Szpakowicz S. Beyond Accuracy, F-Score and ROC: A Family of Discriminant Measures for Performance Evaluation. In: AI 2006: Advances in Artificial Intelligence. Springer Berlin Heidelberg: Berlin, Heidelberg, 2006.

[34] Boughorbel S , Jarray F, El-Anbari M. Optimal classifier for imbalanced data using Matthews correlation coefficient metric. *PLoS One*. 2017;12:e0177678. 28574989 10.1371/journal.pone.0177678 PMC5456046

[35] Marrocco C , Duin RPW, Tortorella F. Maximizing the area under the ROC curve by pairwise feature combination. *Pattern Recogn*. 2008;41:1961–74.

[36] Wang Z , Li J, Chambers A et al. Rapid structure-based annotation and profiling of dihydrochalcones in star fruit (*Averrhoa carambola*) using UHPLC/Q-Orbitrap-MS and molecular networking. *J Agric Food Chem*. 2021;69:555–67. 33356228 10.1021/acs.jafc.0c07362

[37] Picart-Armada S , Fernández-Albert F, Vinaixa M et al. FELLA: an R package to enrich metabolomics data. *BMC Bioinformatics*. 2018;19:538. 30577788 10.1186/s12859-018-2487-5 PMC6303911

[38] Kanehisa M , Goto S. KEGG: Kyoto encyclopedia of genes and genomes. *Nucleic Acids Res*. 2000;28:27–30. 10592173 10.1093/nar/28.1.27 PMC102409

[39] Peng T , Kang JL, Xiong XT et al. Integrated transcriptomics and metabolomics analyses provide insights into the response of Chongyi wild mandarin to *Candidatus* Liberibacter asiaticus infection. *Front Plant Sci*. 2021;12:748209. 34721476 10.3389/fpls.2021.748209 PMC8551615

[40] Wei X , Mira A, Yu QB et al. The mechanism of citrus host defense response repression at early stages of infection by feeding of *Diaphorina citri* transmitting *Candidatus* Liberibacter asiaticus. *Front Plant Sci*. 2021;12:635153. 34168662 10.3389/fpls.2021.635153 PMC8218908

[41] Killiny N , Nehela Y. Metabolomic response to Huanglongbing: role of carboxylic compounds in *Citrus sinensis* response to '*Candidatus* Liberibacter asiaticus' and its vector. *MPMI*. 2017;30:666–78. 28510485 10.1094/MPMI-05-17-0106-R

[42] Hung WL , Wang Y. A targeted mass spectrometry-based metabolomics approach toward the understanding of host responses to Huanglongbing disease. *J Agric Food Chem*. 2018;66:10651–61. 30220206 10.1021/acs.jafc.8b04033

[43] Ma WX , Pang ZQ, Huang XE et al. Citrus Huanglongbing is a pathogen-triggered immune disease that can be mitigated with antioxidants and gibberellin. *Nat Commun*. 2022;13:529. 35082290 10.1038/s41467-022-28189-9 PMC8791970

[44] Hijaz FM , Manthey JA, Folimonova SY et al. An HPLC-MS characterization of the changes in sweet orange leaf metabolite profile following infection by the bacterial pathogen *Candidatus* Liberibacter asiaticus. *PLoS One*. 2013;8:e79485. 24223954 10.1371/journal.pone.0079485 PMC3818228

[45] Xue AH , Liu YQ, Li HX et al. Early detection of Huanglongbing with EESI-MS indicates a role of phenylpropanoid pathway in citrus. *Anal Biochem*. 2021;639:114511. 34883070 10.1016/j.ab.2021.114511

[46] Indrakumari R , Poongodi T, Khaitan S et al. A review on plant diseases recognition through deep learning, in Handbook of Deep Learning in Biomedical Engineering 1st edn. In: Balas VE, Mishra BK, Kumar R, eds. Elsevier Science: Amsterdam, 2021.

[47] Ferentinos KP . Deep learning models for plant disease detection and diagnosis. *Comput Electron Agric*. 2018;145:311–8.

[48] Ma JC , Du KM, Zheng FX et al. A recognition method for cucumber diseases using leaf symptom images based on deep convolutional neural network. *Comput Electron Agric*. 2018;154:18–24.

[49] Schumann A , Waldo L, Mungofa P et al. Computer tools for diagnosing citrus leaf symptoms (part 2): smartphone apps for expert diagnosis of citrus leaf symptoms. *EDIS*. 2020;2020:SL478.

[50] Mujika KM , JaJ M, De Miguel AF. Advantages and disadvantages in image processing with free software in radiology. *J Med Syst*. 2018;42:36. 29333590 10.1007/s10916-017-0888-z

