# Supplementary material for: Nontargeted metabolomics-based multiple machine learning modeling boosts early accurate detection for citrus Huanglongbing
Source: Hortic Res. 2022 Jun 27;9:uhac145. doi: 10.1093/hr/uhac145 (PMC9433982; doi:10.1093/hr/uhac145)

Supplementary Information

**Nontargeted metabolomics-based multiple** **machine learning modeling boosts** **early** **accurate detection for citrus Huanglongbing**

**Zhixin Wang^1^, Yue Niu^2^, Tripti Vashisth^1^, Jingwen Li^1^, Robert Madden^1^, Taylor Shea Livingston^1^, Yu Wang^1,^***

^1^ Citrus Research & Education Center, Institute of Food and Agricultural Sciences, University of Florida, Lake Alfred, Florida 33850-2299, U.S.A.

^2^ Department of Mathematics, University of Arizona, Tucson, Arizona 85721-0089, U.S.A.

**Figure S1.** ROC curves and mean AUCs (%) from 12-fold cross-validation for six machine learning models based on datasets acquired by C_18_ column–negative ion mode (CN)

**Figure S2.** ROC curves and mean AUCs (%) from 12-fold cross-validation for six machine learning models based on datasets acquired by C_18_ column–positive ion mode (CP)

**Figure S3.** ROC curves and mean AUCs (%) from 12-fold cross-validation for six machine learning models based on datasets acquired by HILIC column–negative ion mode (HN)

**Figure S4.** ROC curves and mean AUCs (%) from 12-fold cross-validation for six machine learning models based on datasets acquired by HILIC column–positive ion mode (HP)

**Figure S5.** Mean confusion matrices of 12-fold cross-validation for LR-L2 models based on datasets of top-ranked and annotated features from four acquisition sources

**Figure S6.** Mean confusion matrices of 12-fold cross-validation for GBDT models based on datasets of top-ranked and annotated features from four acquisition sources

**Appendix S1.** An example of external test using ML predictors for early detection of HLB

**Data S1.** qPCR test results of budwoods used for grafting and leaf samples

**Data S2.** All the annotated top-ranked metabolite features with related information

**Data S3.** Up-regulated or down-regulated changes of differential metabolites in enriched pathways


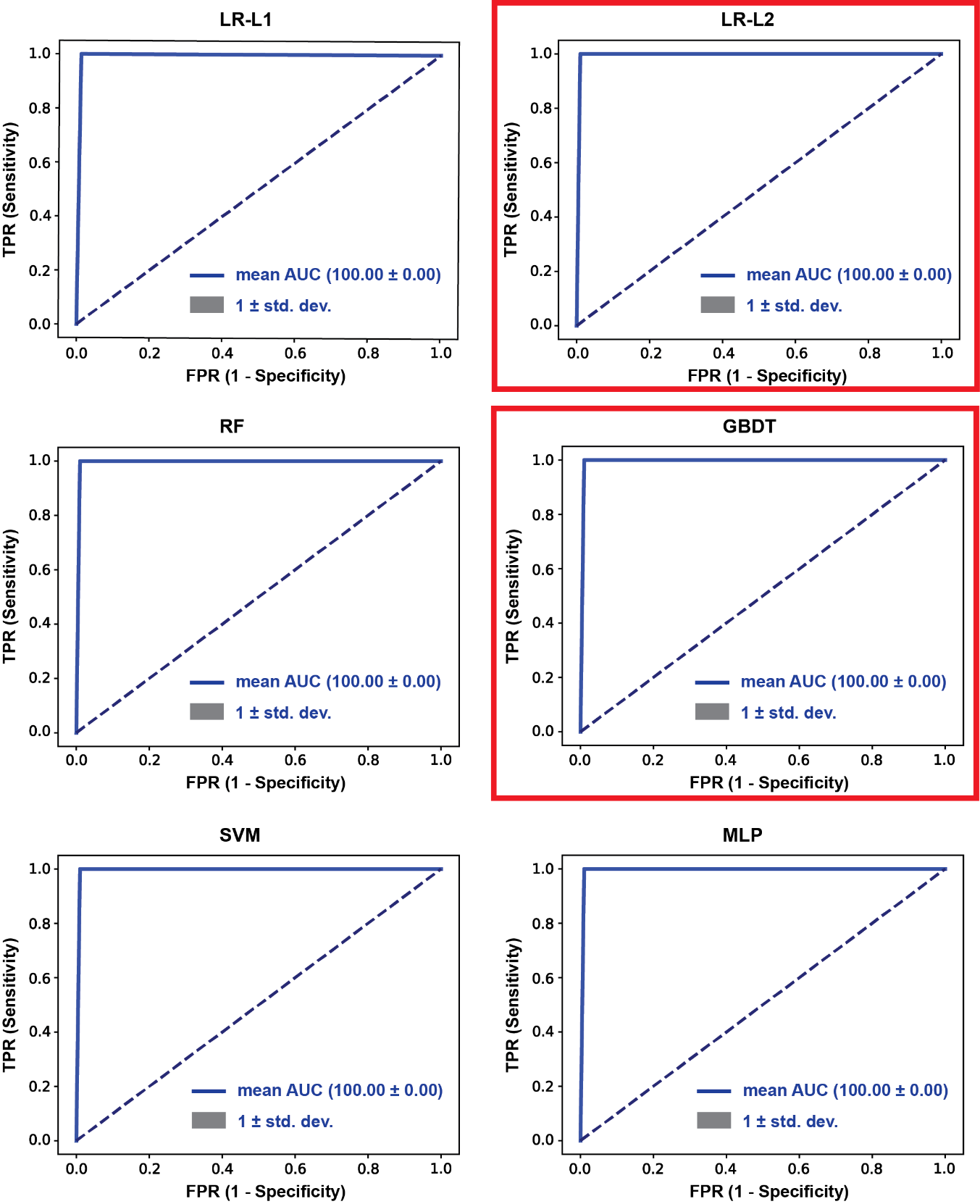


**Figure S1.** ROC curves and mean AUCs (%) from 12-fold cross-validation for six machine learning models based on datasets acquired by C_18_ column–negative ion mode (CN)

**
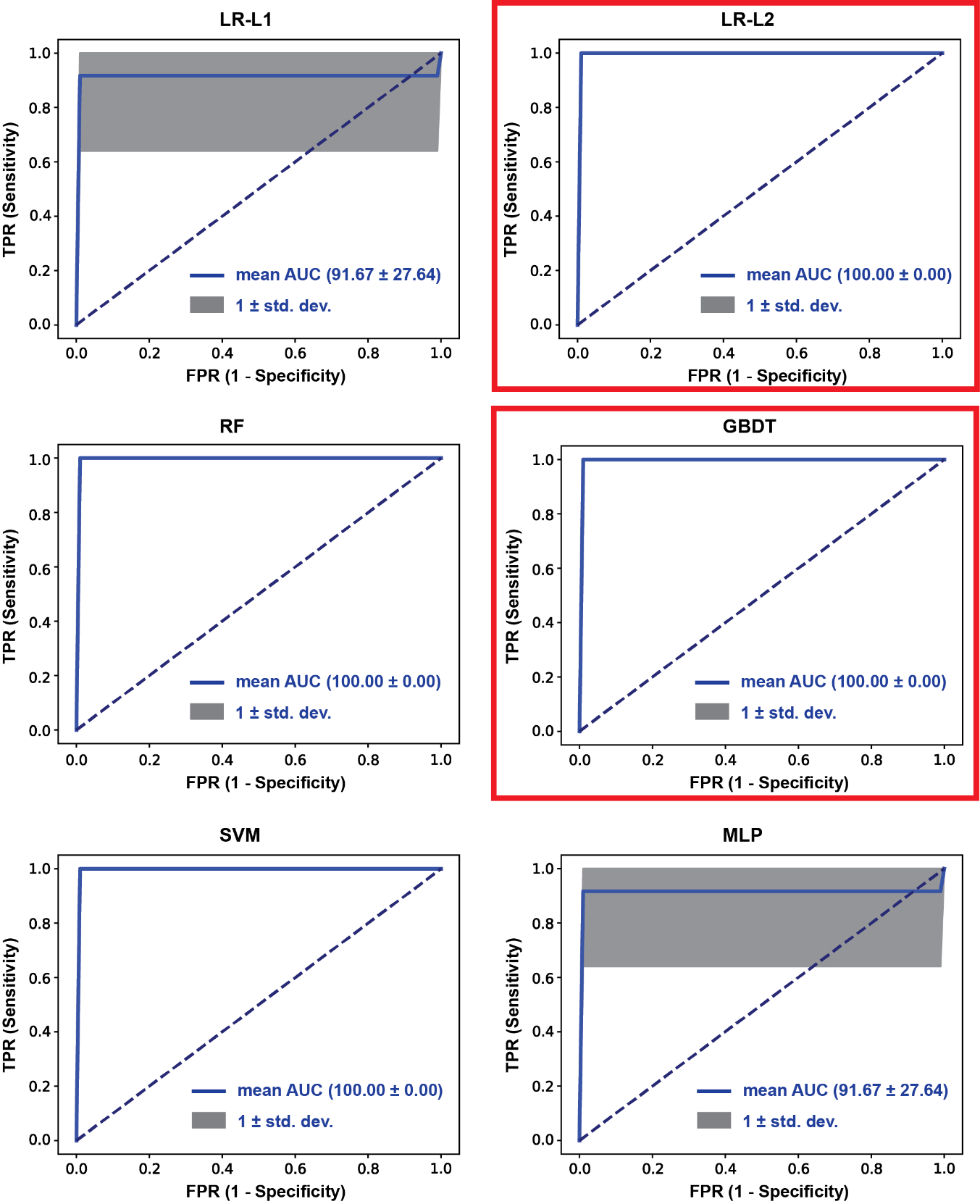
**

**Figure S2.** ROC curves and mean AUCs (%) from 12-fold cross-validation for six machine learning models based on datasets acquired by C_18_ column–positive ion mode (CP)

**
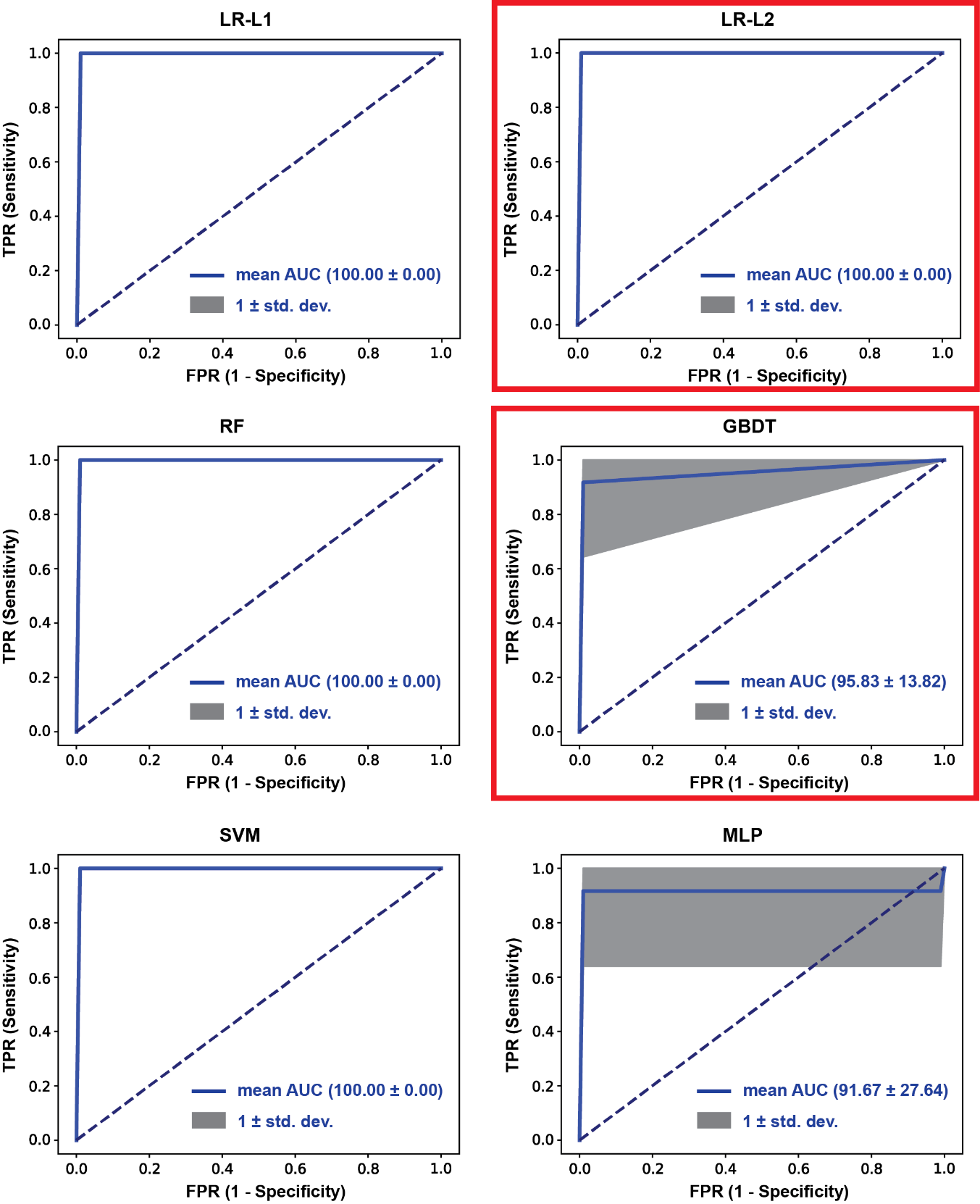
**

**Figure S3.** ROC curves and mean AUCs (%) from 12-fold cross-validation for six machine learning models based on datasets acquired by HILIC column–negative ion mode (HN)

**
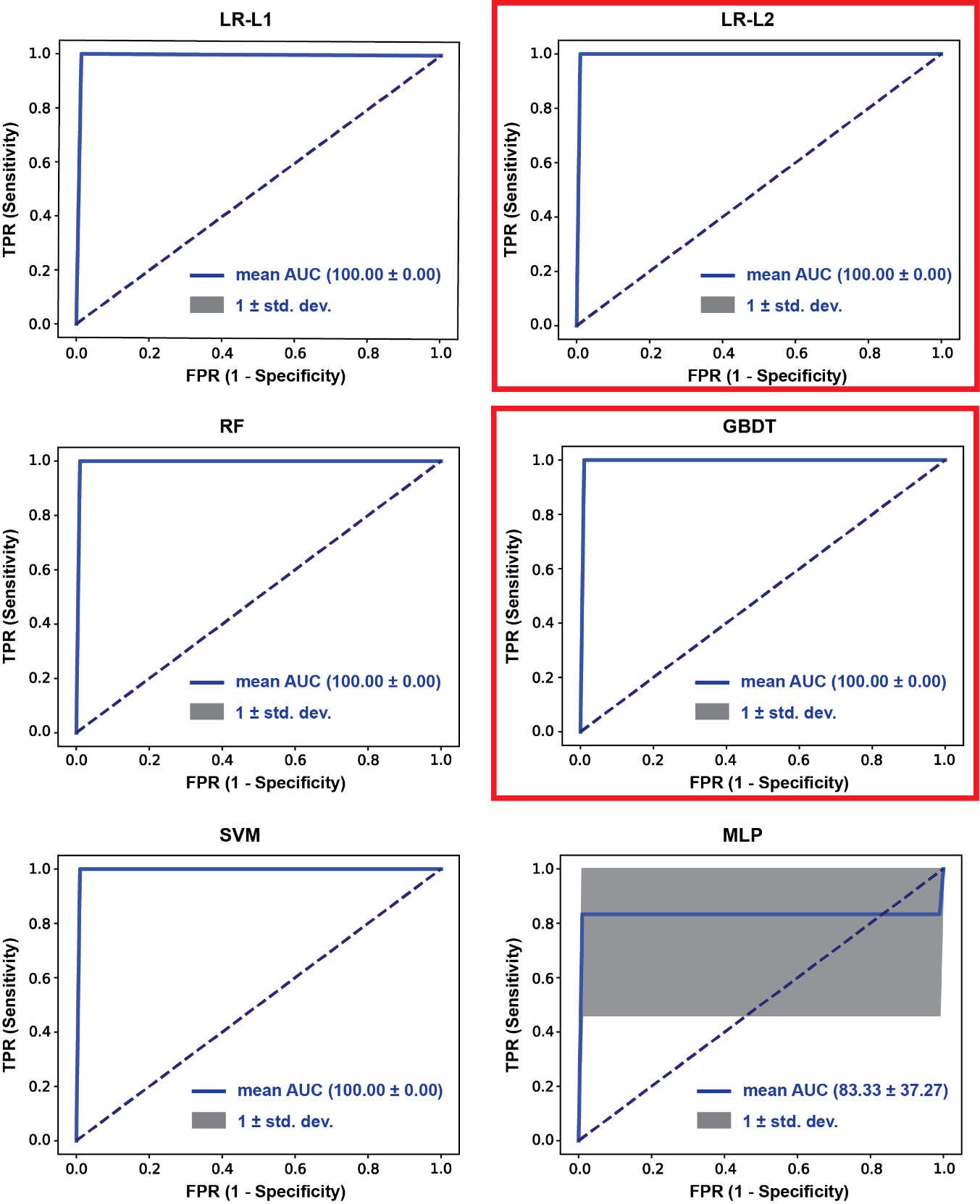
**

**Figure S4.** ROC curves and mean AUCs (%) from 12-fold cross-validation for six machine learning models based on datasets acquired by HILIC column–positive ion mode (HP)

**
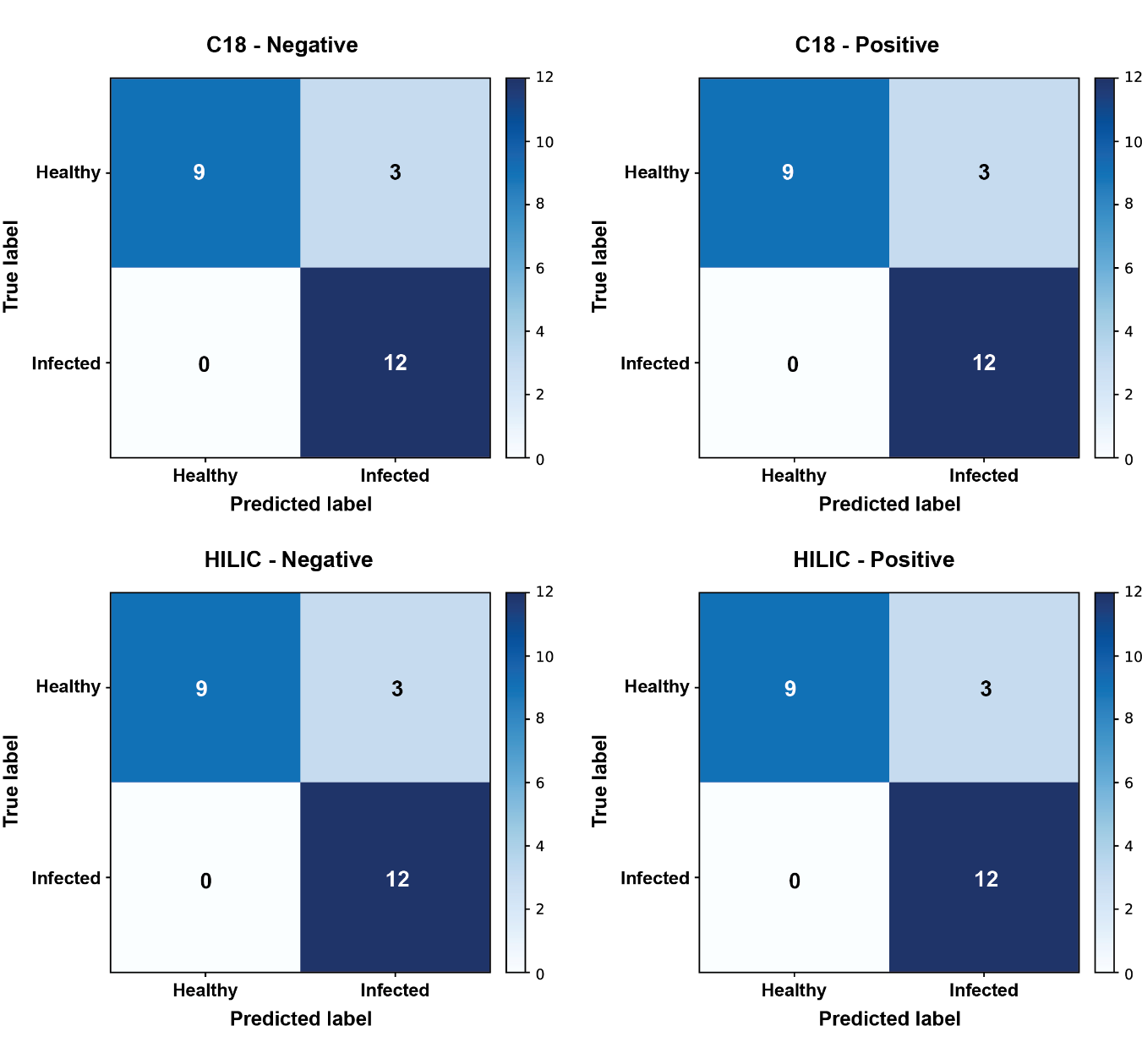
**

**Figure S5.** Mean confusion matrices of 12-fold cross-validation for LR-L2 models based on datasets of top-ranked and annotated features from four acquisition sources

**
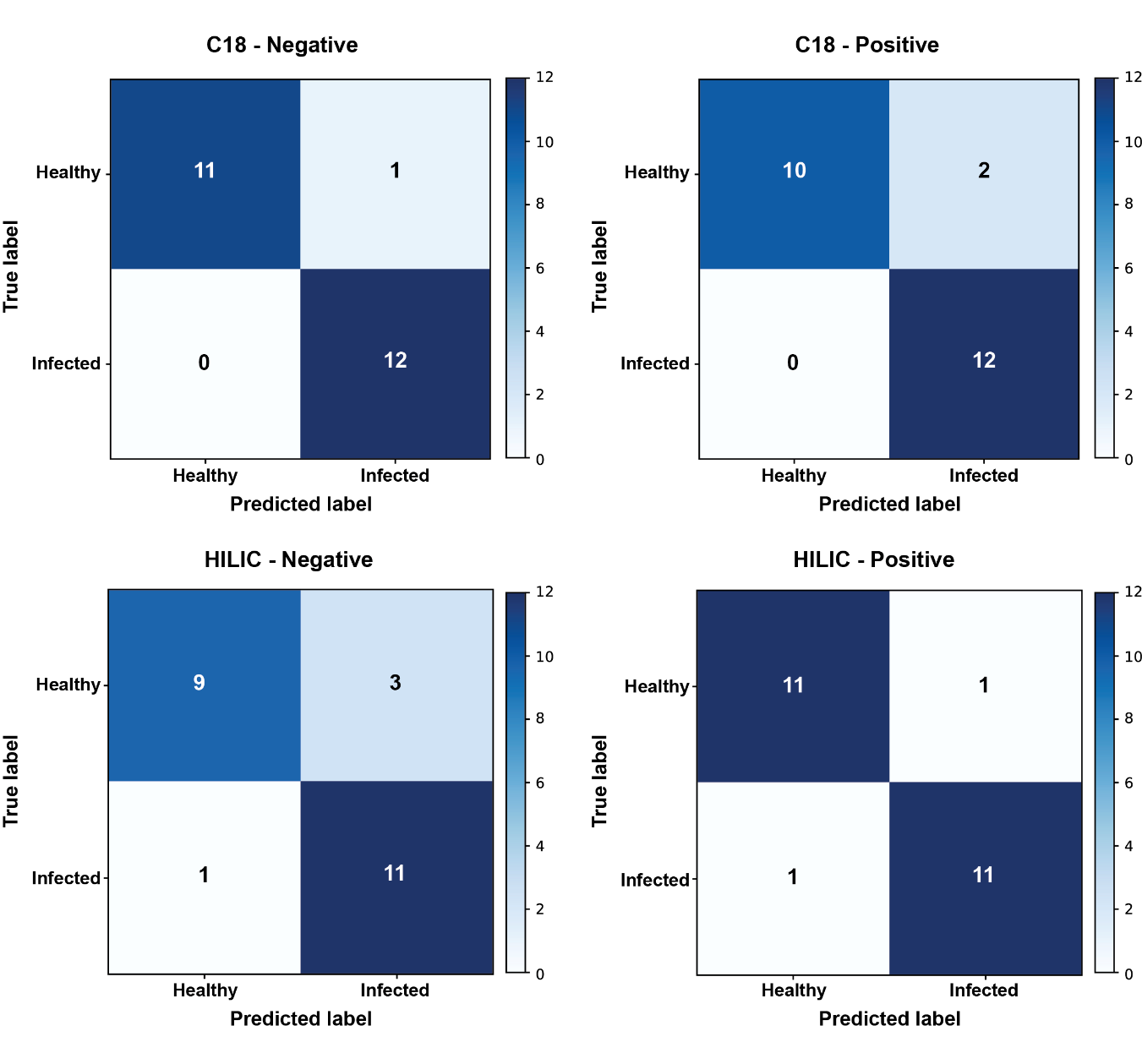
**

**Figure S6.** Mean confusion matrices of 12-fold cross-validation for GBDT models based on datasets of top-ranked and annotated features from four acquisition sources

**Appendix S1.** An example of external test using ML predictors for early detection of HLB

Six additional leaf samples, including three HLB-affected ones and three healthy ones, derived from the same source as those used for modeling, were adopted for the external test. The data were acquired from C_18_ column and negative ion mode (CN), and the settings for instrumentation and data pre-processing were identical as described in the section “Materials and methods”. For each sample, the peak areas of the 108 metabolic biomarkers were selected out as the test data, followed by Z-score normalization. After re-generating the confirmed LR-L2 model and GBDT model with those 108 features, the test data were loaded for HLB detection, respectively. It was shown by the result that the predicted labels for all the six test samples were correct using either the LR-L2 predictor or the GBDT predictor. The accuracies were 100%.

The interested communities can refer to this protocol to perform the early detection of HLB for their samples. Any one of the four predictors (CN/CP/HN/HP) reported in the article can be applied individually or combinedly according to the practical conditions. The metabolic biomarkers included in the four predictors (see Supplementary Data S3) should be taken as the targeted compounds, of which peak areas (or abundances) could be quantified by any available LC-MS instrument methods, such as the selected ion monitoring (SIM) on Ion Trap MS or multiple reaction monitoring (MRM) on QQQ MS.

The datasets and script of Python codes used for multiple ML modeling and external test refer to the GitHub website (<https://github.com/Yu-Wang-Lab/Multiple_ML_modeling_for_HLB_prediction>).

**True labels and predicted results of external test using six samples:**

| **Sample** | **True label** | **LR-L2** | | **GBDT** | |
| --- | --- | --- | --- | --- | --- |
|  |  | **Predicted label** | **HLB-affected probability (%)** | **Predicted label** | **HLB-affected probability (%)** |
| I-13 | infected | infected (1) | 96.47 | infected (1) | 99.88 |
| I-14 | infected | infected (1) | 98.48 | infected (1) | 99.88 |
| I-15 | infected | infected (1) | 99.75 | infected (1) | 99.88 |
| H-13 | healthy | healthy (0) | 0.85 | healthy (0) | 0.12 |
| H-14 | healthy | healthy (0) | 1.17 | healthy (0) | 1.98 |
| H-15 | healthy | healthy (0) | 0.81 | healthy (0) | 1.12 |

**Codes, input and output in console demonstrating external test:**


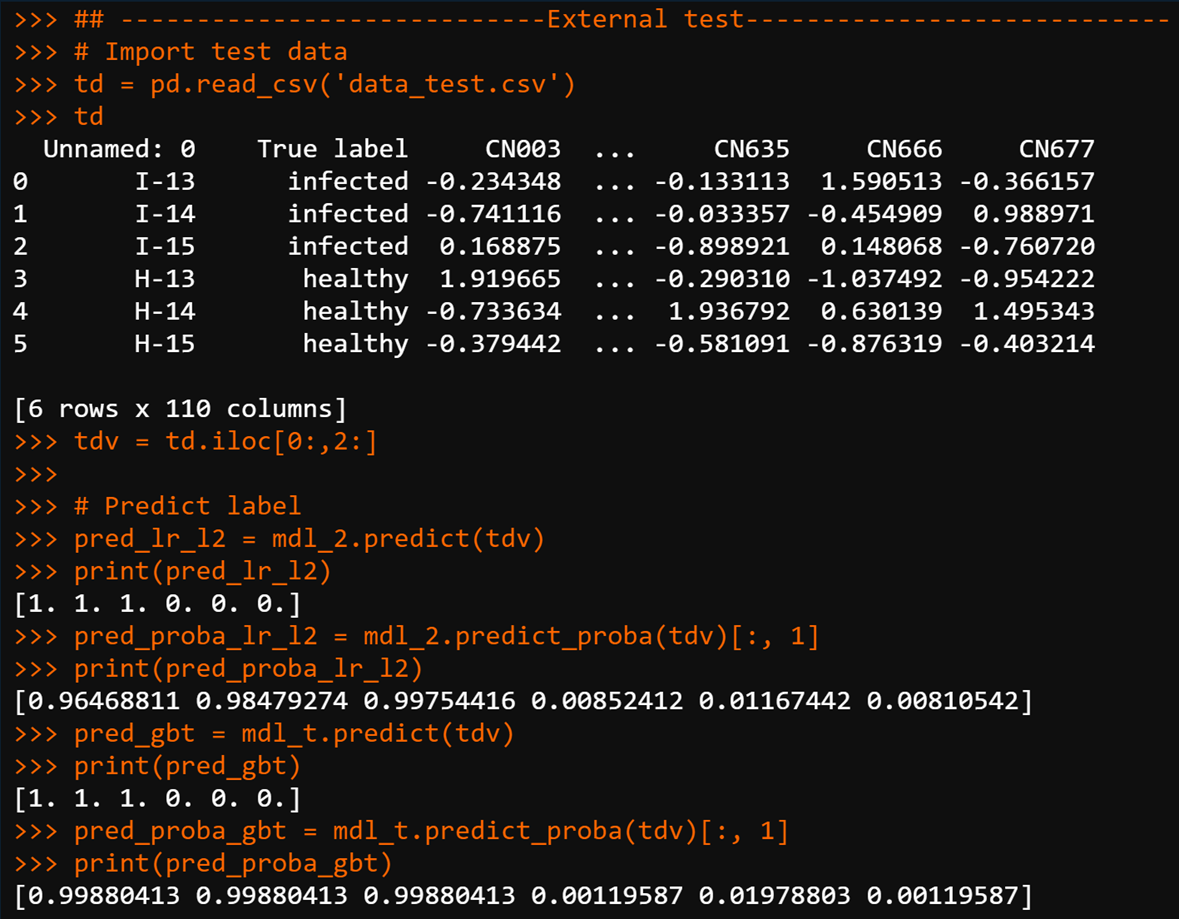

Supplement: Web_Material_uhac145 [file web_material_uhac145.zip › Supplementary Figures S1 to S6 & Appendix S1.docx]
